# The cost of mass drug administration for trachoma in two counties of the Republic of South Sudan

**DOI:** 10.1371/journal.pgph.0003242

**Published:** 2024-07-19

**Authors:** Tim Jesudason, Angelia M. Sanders, Stephen Ohidor, Alexis S. Delahunt, Andrew R. Deathe, Lochebe Boniface, Isaiah Buot, Mekete Bikis, Samual Makoy, Yak Yak Bol, James Niquette, E. Kelly Callahan, Damien Walker, Scott D. Nash

**Affiliations:** 1 Partners in Global Health Ltd, Dereham, United Kingdom; 2 The Carter Center, Atlanta, Georgia, United States of America; 3 The Carter Center, Juba, Republic of South Sudan; 4 The Ministry of Health, Juba, Republic of South Sudan; 5 Independent Contractor, Arlington, VA, United States of America; RTI International, UNITED STATES OF AMERICA

## Abstract

Community-wide distribution of azithromycin, otherwise known as mass drug administration (MDA), is a component of the World Health Organization-endorsed SAFE strategy for the elimination of trachoma as a public health problem. In the Republic of South Sudan, 2.9 million people are known to live in areas that are known to require interventions and warrant MDA. This study estimated the total costs and cost per person treated during MDA in two counties, Kapoeta North and Kapoeta East, in South Sudan. MDA was conducted in Kapoeta North and Kapoeta East counties from October 2020 to January 2021. Following training and community sensitization, a core team, consisting of a height measurer, a drug dispenser, and a data recorder, delivered the intervention. A detailed costing database was developed in Microsoft Excel. An ingredients approach was used to capture all financial and economic costs incurred from a payer perspective. Primary outcomes included the total cost of MDA in each county and the cost per person treated in each county. In Kapoeta North, 58,226 people were treated at a financial cost of $71,350 USD. This corresponds to a unit cost of $1.22 per person treated. The total economic cost of the intervention was $99,036, at a unit cost of $1.70 per person treated. In Kapoeta East, 156,092 people were treated at a total financial cost of $168,404. This corresponds to a unit cost of $1.08 per person treated. The total economic cost of the intervention was $243,205, at a unit cost of $1.56 per person treated. The study highlights the cost variation for MDA implementation across two counties of South Sudan. As the South Sudan trachoma program expands, this information will be valuable for program planning.

## Introduction

The World Health Organization (WHO) has endorsed the SAFE (surgery, antibiotics, facial cleanliness, and environmental improvement) strategy to eliminate trachoma, the world’s leading infectious cause of blindness, as a public health problem [1]. The ‘A’ of the SAFE strategy is composed of community-wide mass drug administration (MDA) with antibiotics. Typically, this includes annual treatment of all residents living in a trachoma endemic district.

MDA for trachoma and other neglected tropical diseases (NTDs) amenable to preventive chemotherapy (PC-NTDs) is widely recognized as a safe, effective, and highly cost-effective intervention in global health [2–4]. Many advocating for investment in PC-NTDs, including trachoma, have cited a figure of around $0.50 USD per person treated per year [5, 6]. However, there is little empirical evidence to support this figure, with published literature demonstrating significant variation in the costs of MDA for trachoma [3, 7, 8].

To achieve the elimination of trachoma as a public health problem by 2030, as targeted by the global road map for NTDs, published by WHO in 2021 [9], up-to-date and accurate costing data are needed to effectively plan programs and advocate for investment. Furthermore, there is a need to understand the cost of implementing MDA in difficult operating environments. This will be particularly important in settings that are hard to reach, due to geographical, environmental, and security factors, which can further affect implementation and increase costs. Previous estimates of the cost of implementation for trachoma MDA in South Sudan have shown that, based on 2010 data, around $1.50 should be budgeted for each person treated [10]. However, the national trachoma program has evolved significantly since 2010, and further investigation of MDA costs is needed to understand how the cost of MDA varies and to identify the main causes of this variation.

**Fig 1:**
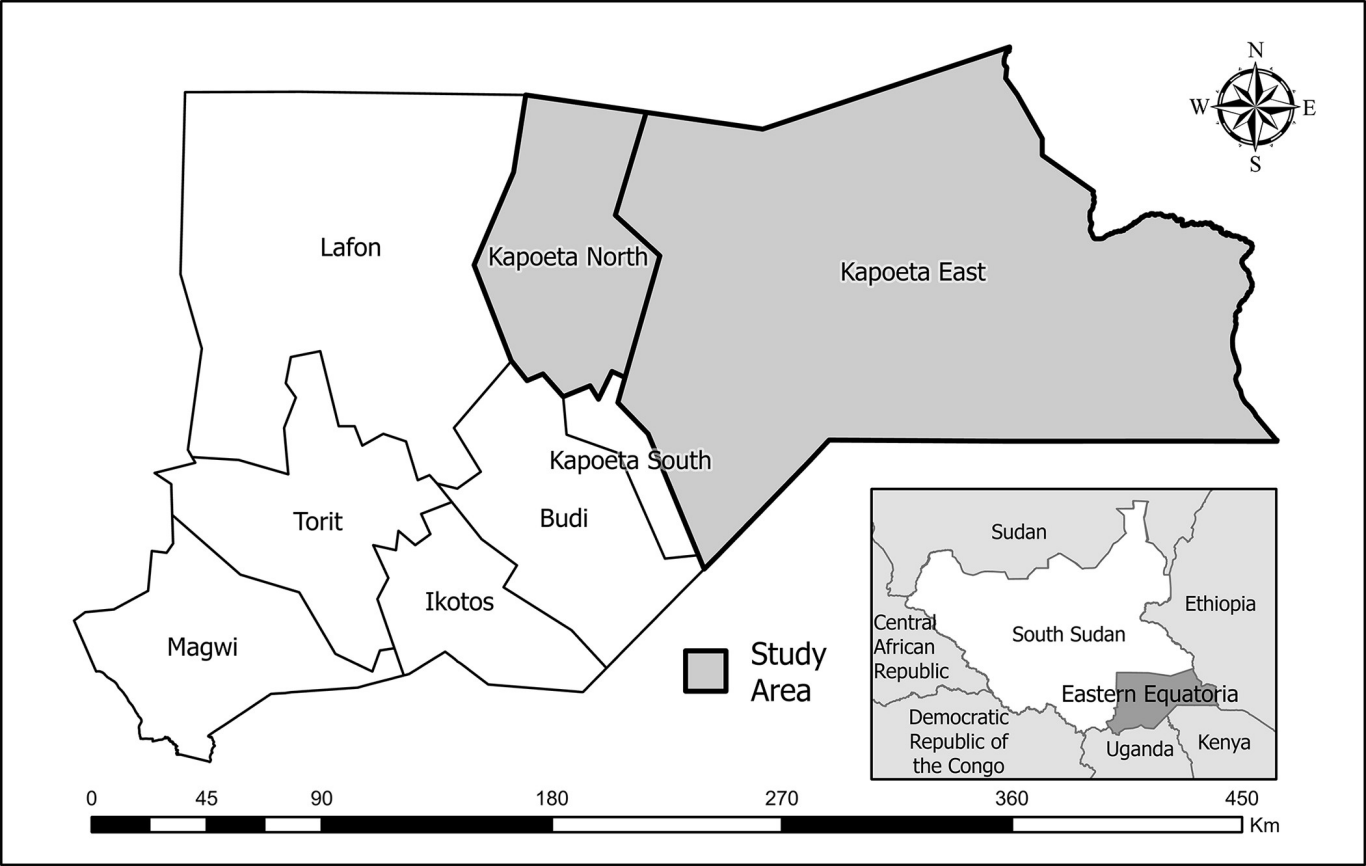
Map of Kapoeta East and Kapoeta North counties, South Sudan. Map created in ArcGIS Pro 2.2.6 (ESRI, Redlands, CA) using a shapefile sourced from the GADM database (gadm.org).

The South Sudan Ministry of Health began implementing MDA in five counties (the equivalent of a district) in Eastern Equatoria State, South Sudan, in 2007. Two of these counties, Kapoeta North and Kapoeta East, are still known to be hyper-endemic for trachoma based on prevalence surveys conducted in 2015 [11] (Fig 1). Both counties are known to be difficult to implement programs in due to highly variable environmental conditions which can affect implementation time. During the rainy season, implementation can be delayed by flooding or difficulties traversing roads to reach village distribution points. On the other hand, during the dry season, portions of the community travel with their cattle to grazing lands known as ‘cattle camps’. This can impact MDA treatment coverage since some people from one county, such as Kapoeta North, will be temporarily residing in cattle camps in another county, such as Kapoeta East.

Given that these environmental conditions and migration issues are common across South Sudan, this study estimated the total cost and the cost per person of MDA conducted between October 2020 to January 2021 in Kapoeta North and Kapoeta East counties as a basis for understanding possible costs for trachoma MDA treatments across South Sudan.

## Methods

### MDA implementation

The WHO-endorsed SAFE strategy recommends the implementation of MDA for all districts (usually with populations of 100,000 to 250,000) in which the prevalence of the active trachoma sign “trachomatous inflammation-follicular” (TF) is ≥ 5% among children ages 1 to 9 years. In those districts, all residents should be offered antibiotic treatment annually, with the planned number of annual rounds depending on the most recent estimate of TF prevalence [1].

The Zithromax Management Guide, published by the International Trachoma Initiative, recommends that individuals ages ≥ 15 years receive an adult dose of four tablets; individuals taller than 120 cm, individuals ages ≥ 7 years to < 15 years receive 3–4 tablets (dosage according to height); all children ≥ 6 months to < 7 years, anyone under 120 cm, or anyone with difficulties swallowing tablets or uncomfortable taking tablets should receive powder for oral suspension (POS; dosage according to height); children ages 0 to < 6 months should receive tetracycline eye ointment (TEO) [2].

In South Sudan, trachoma MDA teams include a height measurer, a drug dispenser, and a data recorder, all of whom receive a two-day training before MDA begins. These teams are supported by supervisors that come from the Ministry of Health County Health Department and The Carter Center, a non-governmental organization financially and technically supporting the Ministry of Health’s Trachoma Control Program in select counties of South Sudan. In most settings, a central drug distribution point is used within the community, followed by house-to-house distributions for those individuals who are unable to leave their houses. Teams typically spend one to two days in each community. Eligible populations are treated according to national guidelines, which are aligned with the International Trachoma Initiative Zithromax Management Guide [2]. The length of the MDA campaign depends on the number of communities, geographical distribution of the communities, the number of teams, and the time of year. The target population for the MDAs are based on the previous year’s MDA treatment numbers with an assumed overall population size increase of 2%. Within South Sudan children 1 to 7 years make up approximately 30% of the population.

Between October 2020 and January 2021, MDA campaigns were conducted in the counties of Kapoeta North and Kapoeta East. Due to the prevalence of active trachoma in these counties, this was the fifth round of MDA conducted since the 2015 prevalence survey.

In Kapoeta North, 10 distribution teams (composed of a total of 30 people) were deployed for 17 days between November 21, 2020, and December 8, 2020. The total population treated was 58,226 out of a target population of 69,102 (Table 1). Of these, 22,826 received Zithromax POS, 31,484 received Zithromax tablets, and 3,916 received TEO. Overall, 84% coverage of the targeted population was achieved. The number of treatments by payam (subdistrict) and the number of villages in each payam is presented in Supporting Information Table 1 (S1 Table).

**Table 1:** Summary of Kapoeta North and Kapoeta East MDA achievements, South Sudan, 2020.

County	Total population treated	Target population	Coverage rate
**Kapoeta North**	58,226	69,102	84%
**Kapoeta East**	156,092	151,154	103%

In Kapoeta East, 51 distribution teams (composed of a total of 153 people) were deployed at various time points between October 30, 2020, and January 26, 2021. Due to the larger geographical size of the county, teams were divided into five sub-areas and implementation took between five and 17 days in each sub-area (S2 Table). The total population treated in Kapoeta East was 156,092 out of a target population of 151,154. Of the total number of people treated, 52,474 received Zithromax POS, 92,239 received Zithromax tablets, and 11,379 received TEO. Overall, 103% coverage of the targeted population was achieved.

To reduce the risk of transmission of COVID-19 during MDA trainings, the national program conducted trainings outside, kept attendees six feet apart, required face masks and provided hand sanitizer. During implementation of the MDA, the national program required drug distribution teams to wear face masks, carry and use hand sanitizer at all distribution sites, required families to provide their own cup of water to use when swallowing tablets, and adjusted the drug distribution method to be house to house, as compared to a central location that, prior to COVID-19, entailed all villagers coming to a central location and lining up to receive drug. Because drug distributors are recruited within the counties where MDA was implemented and because of the layout of households within villages, no additional costs were required to guide distribution teams during house-to-house administration.

### Costing methods

This study followed best practices as stated in the Global Health Cost Consortium Reference Case [12] and utilized the Global Health Cost Consortium Principles and Methods Checklist to ensure methodological alignment [13].

A detailed costing database was developed in Microsoft Excel. An ingredients approach was used to capture all financial and economic costs. The costs were estimated from a payer perspective and encompassed the real-world implementation costs incurred by The Carter Center. The county was the unit of analysis.

Primary outcomes included the total cost of MDA in each county and the cost per person treated in each county. Secondary outcomes included the cost per person treated at different MDA coverage levels. Societal costs, such as productivity losses and participant costs incurred, were not included.

Financial costs capture the resources that are paid for, and thus, do not include donated goods, such as medicines. Accordingly, financial costs included all cash expenditures required to implement the MDA campaign. The financial costs of capital items were identified using straight-line depreciation (dividing the total cost by the years of useful life) [12], followed by a calculation of the proportion of capital items attributable to activities in Kapoeta North and Kapoeta East (S3 Table). A daily rate for the use of capital costs such as vehicles and salaries were not applied, as these costs are incurred primarily for the implementation of MDA activities. Therefore, an annualized proportion of costs was deemed appropriate to avoid underestimating the cost of capital items.

Economic costs aim to capture opportunity costs or the value of the highest-value alternative health intervention opportunity forgone [12]. We calculated the economic costs of all resources required for the campaign. This provides a more comprehensive estimate than capturing the financial costs because it captures the economic value of all resources, including the opportunity costs of donated drugs and equipment that did not incur any costs to The Carter Center. Consequently, the economic costs are always higher than the financial costs of the program. We included a proportion of the costs of capital items with a value over $100 and an expected useful life of more than one year. Capital items were annualized and discounted over their estimated useful life using a discount rate of 3% as recommended by the Global Health Cost Consortium [12].

Average exchange rates for the period 1 January to 31 December 2020 were used for currency conversions, as provided by https://cuex.com/. The rates were: $1   =  130.26 South Sudanese Pounds. Research costs associated with this costing study were not included in the analysis.

### Cost activities

Table 2 presents MDA activities included in the study and the associated cost categories. The quantity and price of all resources were identified to evaluate total intervention costs (S4 Table). All costs were identified retrospectively through a review of The Carter Center’s financial documents and, when required, through discussion with program staff. To ensure costs such as salaried labor were not double counted, we assumed these and other capital expenditures to be central program costs. These costs were not divided into specific MDA activities, as the costs are required centrally to implement MDA.

**Table 2:** Cost activities and ingredients in Kapoeta North and Kapoeta East, South Sudan, 2020.

Input/line item	Training	Community sensitization	Drug transportation	Drug administration	Central program costs
**Office costs**					X
**Communication and IT**					X
**Accommodation Equipment**					X
**Vehicles**					X
**Other capital**					X
**Salaried labor**					X
**Per diems**	X	X	X	X	
**Zithromax tablets**				X	
**Powder oral suspension**				X	
**Tetracycline eye ointment**				X	
**Utilities**					X
**Security**					X
**Transport/fuel**	X	X	X	X	
**Vehicle maintenance**					X
**Printing**	X	X		X	X

In instances where unit costs and quantities were unavailable, we applied gross costs or used a top-down approach and divided the total cost of expenditure and applied the cost across the relevant categories. Most notably, the cost of fuel was divided proportionately to the number of days allocated to training, community sensitization, drug administration, and supervision. Gross costs included materials purchased for attendee training kits, such as stationary, and MDA form printing, such as distribution logs and certificates.

Central program costs, including staff salaries and capital items, such as vehicles, laptops, and satellite phones, were considered central costs that benefited all trachoma activities coordinated by the Trachoma Control Program. As such, only a proportion of central program costs were attributed to MDA activities in Kapoeta North and Kapoeta East. To estimate the proportion of costs, we used the relative population of Kapoeta North and Kapoeta East. This was done by calculating the proportion of the target population of Kapoeta North and Kapoeta East against the entire target population of the five counties in Eastern Equatoria State that the central program infrastructure in South Sudan supported in 2020. Program staff estimated that 75% of central program costs were allocated to MDA activities in 2020.

### Sensitivity analyses

We applied univariate sensitivity analyses to explore how program changes affect the primary outcomes. Univariate sensitivity analyses allow for manually changing one variable/input parameter of interest while keeping all other variables in the model constant [13, 14]. We explored the following key parameters: 1) increased the useful lifespan of capital items. This included simultaneously increasing the average life of a vehicle from 10 to 15 years, increasing the average life of a motorbike from five to eight years, increasing the average life of a laptop and satellite phone from five to eight years, and increasing the average life of a tent from two to three years; 2) we reduced the time to deliver interventions to 10 days to show the cost at a relatively fast rate of implementation; 3) we adjusted the number of people treated while holding the number of treatment days constant to calculate the cost per person treated at 80%, 90% and 100% coverage; 4) we analyzed the cost of MDA if an equal amount of central program costs were allocated to MDA in Kapoeta North and Kapoeta East. In doing so, we changed the proportion of central program costs attributed to Kapoeta North MDA from 19% to 20%; we also changed the proportion of central program costs associated with Kapoeta East from 42% to 20%; and 4) we removed all central program costs to calculate the additional cost that should be budgeted to implement MDA in each county after salaries, capital equipment and other office costs are accounted for. The map of the study areas was created in ArcGIS Pro 2.2.6 (ESRI, Redlands, CA) using shapefiles sourced from the GADM database (gadm.org).

## Ethics statement

MDA activities were conducted in collaboration with the Ministry of Health, South Sudan. The MDA was conducted in alignment with the International Trachoma Initiative Zithromax Management guide [2]. The costing study included only data on expenditures and resources used by The Carter Center in the implementation of the campaign. Data collection did not involve human participants and therefore did not require ethical approval. Additional information regarding the ethical, cultural, and scientific considerations specific to inclusivity in global research is included in the supporting information (S1 Checklist).

## Results

### Kapoeta north county

The total financial cost to implement MDA in Kapoeta North was $71,350 (Table 3). The major cost driver for implementation was central program costs, which totaled $49,026 (69% of total costs). Staff salaries accounted for 34% of total costs while capital items accounted for 9% of total costs. The financial cost per person treated was $1.22.

**Table 3:** Financial costs of MDA in Kapoeta North by cost categories, South Sudan, 2020*.

Cost categories	Cost	Proportion of total cost
**Central program costs**	$49,026	69%
**Training**	$1,588	2%
**Community sensitization**	$2,110	3%
**Drug transport**	$2,362	3%
**Drug administration**	$11,702	16%
**Supervision**	$4,563	6%

* Individual line items might not add up to the total due to rounding.

The total economic cost for implementation was $99,036. The major cost driver for implementation was central program costs, which totaled $51,730 (52% of total costs). Staff salaries accounted for 24% of total costs while capital items accounted for 9% of total costs. (Table 4). The economic cost per person treated was $1.70.

**Table 4:** Economic cost of MDA in Kapoeta North by cost categories, South Sudan, 2020*.

Cost categories	Cost	Proportion of total cost
**Central program costs**	$51,730	52%
**Training**	$1,668	2%
**Community sensitization**	$2,150	2%
**Drug transport**	$2,362	2%
**Drug administration**	$36,563	37%
**Supervision**	$4,563	5%

* Individual line items might not add up to the total due to rounding.

### Kapoeta east county

The total financial cost to implement MDA in Kapoeta East was $168,404 (Table 5). The major cost driver for implementation was central program costs, which totaled $110,068 (65% of total costs). Staff salaries accounted for 32% of total costs while capital items accounted for 9% of total costs. The financial cost per person treated was $1.08.

**Table 5:** Financial costs of MDA in Kapoeta East by cost categories, South Sudan, 2020*.

Cost categories	Cost	Proportion of total cost
**Central program costs**	$110,068	65%
**Training**	$4,358	3%
**Community sensitization**	$3,742	2%
**Drug transport**	$5,136	3%
**Drug administration**	$37,756	22%
**Supervision**	$7,343	4%

* Individual line items might not add up to the total due to rounding.

The total economic cost for implementation was $243,205. The major cost driver for implementation was central program costs, which totaled $115,983 (48% of total costs). Staff salaries accounted for 22% of total costs while capital items accounted for 8% of total costs. (Table 6). The economic cost per person treated was $1.56.

**Table 6:** Economic cost of MDA in Kapoeta East by cost categories, South Sudan, 2020*.

Cost categories	Cost	Proportion of total cost
**Central program costs**	$115,983	48%
**Training**	$4,958	2%
**Community sensitization**	$3,782	2%
**Drug transport**	$5,136	2%
**Drug administration**	$106,003	44%
**Supervision**	$7,343	3%

* Individual line items might not add up to the total due to rounding.

### Sensitivity analysis

When assumptions about useful life of capital items were increased, the total financial cost of MDA in Kapoeta North reduced from $71,350 to $69,121. The financial cost per person treated reduced from $1.22 to $1.19. The total economic cost reduced from $99,036 to $96,859. The economic cost per person treated reduced from $1.70 to $1.66. In Kapoeta East, the total financial cost reduced from $168,404 to $163,526. The financial cost per person treated reduced from $1.08 to $1.05. The total economic cost reduced from $243,205 to $238,445. The economic cost per person treated reduced from $1.56 to $1.53.

When the time to implement MDA was adjusted to 10 days, the total financial cost of MDA in Kapoeta North decreased from $71,350 to $67,927. The financial cost per person treated decreased from $1.22 to $1.17. The total economic cost decreased from $99,036 to $95,620. The economic cost per person treated decreased from $1.70 to $1.64. In Kapoeta East, the total financial cost decreased from $168,404 to $163,493. The financial cost per person treated decreased from $1.08 to $1.05. The total economic cost decreased from $243,205 to $238,312. The economic cost per person treated decreased from $1.56 to $1.53.

Based on the targeted population, and holding the number of treatment days constant, the cost per person treated at different coverage levels varied (Table 7). In Kapoeta North the financial cost per person treated was $1.29 at 80% coverage. This reduced to $1.03 with 100% coverage. The economic cost per person treated in Kapoeta North reduced from $1.79 at 80% coverage to $1.43 per person treated at 100% coverage. In Kapoeta East the financial cost per person treated was $1.39 at 80% coverage. This reduced to $1.11 at 100% coverage. The economic cost per person treated in Kapoeta East reduced from $2.01 at 80% coverage to $1.61 per person treated at 100% coverage.

**Table 7:** Cost per person treated at different coverage rates in Kapoeta North and Kapoeta East counties, South Sudan.

Coverage rate	Kapoeta North: Financial cost per person treated	Kapoeta North: Economic cost per person treated	Kapoeta East: Financial cost per person treated	Kapoeta East: Economic cost per person treated
**80%**	$1.29	$1.79	$1.39	$2.01
**90%**	$1.15	$1.59	$1.24	$1.79
**100%**	$1.03	$1.43	$1.11	$1.61

When the percentage of central program costs attributed to Kapoeta North was increased from 19% to 20%, the total financial cost of MDA in Kapoeta North increased from $71,350 to $73,996. The financial cost per person treated increased from $1.22 to $1.27. The total economic cost increased from $99,036 to $101.821. The economic cost per person treated increased from $1.70 to $1.75.

When the percentage of central program costs attributed to Kapoeta East was reduced from 42% to 20%, the total financial cost reduced from $168,404 to $105,398. The financial cost per person treated reduced from $1.08 to $0.68. The total economic cost reduced from $243,205 to $177,121. The economic cost per person treated reduced from $1.56 to $1.13.

When central program costs were considered “sunk” costs and excluded from the analysis, the additional financial cost that should be budgeted to implement MDA in North Kapoeta was $22,324, at a cost per person treated of $0.38. The total economic cost was $47,306 at a cost per person treated of $0.81 Excluding central program costs from the analysis in Kapoeta East, the total financial cost to implement MDA was $58,335, at a cost per person treated of $0.37. The total economic cost was $127,222, at a cost per person treated of $0.82.

## Discussion

This costing analysis found the financial cost of trachoma MDA conducted between October 2020 to January 2021 ranged from $1.08 in Kapoeta East to $1.22 in Kapoeta North. The total economic costs ranged from $1.56 in Kapoeta East to $1.70 in Kapoeta North. Despite Kapoeta East being almost three times larger geographically and containing several mountainous areas and cattle camps which are logistically more time consuming to reach, the cost per person treated in Kapoeta East was lower than Kapoeta North. This was likely due to the larger population treated in Kapoeta East and higher coverage of people treated.

The study suggests that the financial cost of implementing MDA in South Sudan has reduced since the last known costing analysis [10]. The study, published in 2010, found cost of MDA in Mayom county, Unity state, was $1.37 when accounting for financial costs and $1.53 per person treated when accounting for economic costs. During the MDA campaign, 123,760 people were treated at a coverage rate of 94%. Further research would help to identify the cause of cost reductions.

The often-observed economies of scale associated with NTD MDA programs were demonstrated in the sensitivity analysis. Fluctuations in MDA coverage rate will impact the per-person cost of treatment as the total cost is divided by the number of people treated. In South Sudan fluctuations in coverage over different years and counties has been observed, as evidenced in this study which had an 84% coverage rate in Kapoeta North and 103% coverage rate in Kapoeta East. Holding the number of days of the campaign constant and increasing the number of people treated resulted in a relatively small increase in overall financial and economic expenditure but significantly reduced the cost per person treated. In other words, the marginal cost of MDA decreased as the population treated increased as has been observed in other studies [15]. This suggests that cost efficiencies can be achieved as South Sudan continues to scale up its national trachoma MDA activities. However, the results of our sensitivity analysis should be interpreted cautiously as increasing coverage could also increase program costs particularly if the target population is very difficult to reach and would require additional days.

This study suggests that both reducing and increasing the time it takes to conduct an MDA program has a limited impact on the overall costs of MDA. The reason that costs do not reduce significantly is that the driving costs for implementing MDA programs are fixed central program costs, including salaries and capital expenditure. Decreasing units, such as per diems, do not significantly change the cost per person treated when a program takes a longer or shorter time to implement. Interestingly, this study shows that changing the MDA delivery modality from distributing medicines from a central location point to house-to-house distribution likely had a limited impact on the time to implement the program. Therefore, in some contexts, it may be cost effective for a program to take slightly longer to reach a higher number of people and increase coverage than to have a shorter MDA implementation period and reach fewer people.

In our sensitivity analysis, we assessed the additional cost that needs to be budgeted to implement MDA after central program costs, including salaries and capital expenditure, have been accounted for. In this analysis, the financial unit costs ranged from $0.37 to $0.38 and economic unit costs ranged from $0.81 to $0.82 per person treated. This number is similar with commonly cited figures used to advocate for NTDs [5, 6, 16]. As consideration is given to more frequent than annual MDA [17], the additional costs for each additional round of MDA might be like those listed here, if the central program costs are already accounted for as part of a program’s annual budget. However, to our knowledge, there is little information on costing as related to more frequent than annual trachoma MDAs.

This study was limited by uncertainty around the population denominator used by the program for purposes of establishing a target population and measuring MDA coverage. Given that MDAs have been conducted in these two counties for multiple years, treatment targets are typically based on the previous year coverage plus an assumed two percent population growth. Therefore, the coverage rate of 84% in Kapoeta North and 103% in Kapoeta East is uncertain as the denominator was based on this method of establishing targets rather than through a community census. Having a reliable denominator for calculating administrative coverage is a problem that all trachoma programs face [18]. Our analysis of the cost per person treated at varying coverage rates provides an indication of how the unit cost changes because of a changing denominator. This study also did not include economic costs associated with Ministry of Health personnel, as the perspective of the study was to identify costs incurred by The Carter Center in their capacity as a supporting donor and implementing partner. The study did not include the cost of technical support from The Carter Center headquarters in Atlanta, which was determined to be minimal with no staff travel required to implement MDA activities in South Sudan. This study utilized expert opinion from program staff to determine that MDA activities utilized 75% of central program costs, which could introduce recall bias. Improved data collection, including the use of timesheets for program staff, would help reduce this uncertainty in future analyses.

The activities included in this study were conducted during the COVID-19 pandemic and may have resulted in longer implementation time in some communities in both counties. However, in the context of South Sudan, other factors not associated with COVID-19 such as drought, flooding, or insecurity can result in population movement which would also increase the amount of time needed to implement MDA. Therefore, in this context, the results of this analysis are likely applicable to a non-COVID-19 scenario as well. While the results of this study are context specific to Kapoeta North and Kapoeta East, many counties in South Sudan have similar programmatic conditions. Therefore, the findings can still provide a reference point for budgeting.

## Conclusion

Prevalence surveys conducted in South Sudan in 2021 and 2022 demonstrated that at least 31 of the surveyed counties required at least one year of MDA and that some counties required at least three to five rounds [19]. To ensure that the South Sudan trachoma program has the financial resources to conduct the required MDA rounds at high coverage rates, the Ministry of Health and supporting implementing partners may need to consider budgeting MDA at a delivery cost of about $1.22 per person targeted.

## Supporting information

S1 Checklist Inclusivity in global research. (DOCX)

S1 Table Population treated and number of villages reached in Kapoeta East and Kapoeta North counties during 2020 MDA. (DOCX)

S2 Table List of sub-areas and days of implementation in Kapoeta East county, South Sudan. (DOCX)

S3 Table The proportion of capital costs attributable to each county. (DOCX)

S4 Table List of costs and quantities included in costing analysis of MDA in Kapoeta East and Kapoeta North counties, South Sudan. (DOCX)

## References

[1] World Health Organization. WHO Alliance for the Global Elimination of Trachoma: progress report on elimination of trachoma. World Epidemiological Record. 2022;31(97):353–64.

[2] International Trachoma Initiative. Zithromax Management Guide 2019 2019 [cited 2023 February 28, 2023]. Available from: https://www.trachoma.org/zithromax-management-guide.

[3] Frick KD, Lietman TM, Holm SO, Jha HC, Chaudhary JS, Bhatta RC. Cost-effectiveness of trachoma control measures: comparing targeted household treatment and mass treatment of children. Bull World Health Organ. 2001;79(3):201–7. PMC2566378 11285663 PMC2566378

[4] Baltussen R, Smith A. Cost effectiveness of strategies to combat vision and hearing loss in sub-Saharan Africa and South East Asia: mathematical modelling study. BMJ. 2012;344:e615. Epub 2012/03/06. doi: 10.1136/bmj.e615 ; PubMed Central PMCID: PMC3292524. 22389341 PMC3292524

[5] Fenwick A, Molyneux D, Nantulya V. Achieving the Millennium Development Goals. Lancet. 2005;365(9464):1029–30. Epub 2005/03/23. doi: 10.1016/S0140-6736(05)71134-X . 15781095

[6] Brady MA, Hooper PJ, Ottesen EA. Projected benefits from integrating NTD programs in sub-Saharan Africa. Trends Parasitol. 2006;22(7):285–91. Epub 2006/05/30. doi: 10.1016/j.pt.2006.05.007 . 16730230

[7] Schemann JF, Guinot C, Traore L, Zefack G, Dembele M, Diallo I, et al. Longitudinal evaluation of three azithromycin distribution strategies for treatment of trachoma in a sub-Saharan African country, Mali. Acta Trop. 2007;101(1):40–53. Epub 2007/01/24. doi: 10.1016/j.actatropica.2006.12.003 . 17239332

[8] West SK MH, Munoz B, Frick K, editor Provision of Mass Drug Administration: Cost per gain in coverage. ARVO Annual Meeting; 2013: Investigative Opthalmology & Visual Sciences.

[9] World Health Organization. The World Health Organization. Ending the neglect to attain the Sustainable Development Goals: a road map for neglected tropical diseases 2021–2030: World Health Organization; 2021 [cited 2022 14 September]. Available from: https://www.who.int/publications/i/item/9789240010352.

[10] Kolaczinski JH, Robinson E, Finn TP. The cost of antibiotic mass drug administration for trachoma control in a remote area of South Sudan. PLoS Negl Trop Dis. 2011;5(10):e1362. Epub 2011/10/25. doi: 10.1371/journal.pntd.0001362 ; PubMed Central PMCID: PMC3191128. 22022632 PMC3191128

[11] Sanders AM, Stewart AEP, Makoy S, Chebet JJ, Magok P, Kuol A, et al. Burden of trachoma in five counties of Eastern Equatoria state, South Sudan: Results from population-based surveys. PLoS Negl Trop Dis. 2017;11(6):e0005658. Epub 2017/06/15. doi: 10.1371/journal.pntd.0005658 ; PubMed Central PMCID: PMC5484542. 28614375 PMC5484542

[12] Vassall A SS, Kahn J, Gomez GB, Bollinger L, Marseille E, Herzel B, et al. Reference Case for Estimating the Costs of Global Health Services and Interventions. Global Health Cost Consortium 2017;(Available at: https://ghcosting.org/pages/standards/reference_case).

[13] Global Health Cost Consortium. Principles and methods reporting checklist. Global Health Cost Consortium 2017 [cited 2023 February 28, 2023]. Available from: https://ghcosting.org/pages/standards/appendices/principles_and_methods_reporting_checklist.

[14] Deterministic Sensitivity Analysis [online]. York: York Health Economics Consortium; 2016 [cited 2024 June 10, 2024]. Available from: https://yhec.co.uk/glossary/deterministic-sensitivity-analysis/.

[15] Turner HC, Toor J, Hollingsworth TD, Anderson RM. Economic Evaluations of Mass Drug Administration: The Importance of Economies of Scale and Scope. Clin Infect Dis. 2018;66(8):1298–303. Epub 2017/11/11. doi: 10.1093/cid/cix1001 ; PubMed Central PMCID: PMC5888956. 29126255 PMC5888956

[16] Molyneux DH, Hotez PJ, Fenwick A. "Rapid-impact interventions": how a policy of integrated control for Africa’s neglected tropical diseases could benefit the poor. PLoS Med. 2005;2(11):e336. Epub 2005/10/11. doi: 10.1371/journal.pmed.0020336 ; PubMed Central PMCID: PMC1253619 16212468 PMC1253619

[17] World Health Organization. Informal consultation on end-game challenges for trachoma elimination, Task Force for Global Health, Decatur, United States of America, 7–9 December 2021. 2022.

[18] Cromwell EA, Ngondi J, Gatpan G, Becknell S, Kur L, McFarland D, et al. Estimation of population coverage for antibiotic distribution for trachoma control: a comparison of methods. International health. 2009;1(2):182–9. Epub 2009/12/01. doi: 10.1016/j.inhe.2009.09.002 . 24036565

[19] Trachoma Atlas. 2023 [cited 2023 January 11, 2023]. Available from: https://atlas.trachomadata.org/.

