# Supplementary material for: The cost of mass drug administration for trachoma in two counties of the Republic of South Sudan
Source: PLOS Glob Public Health. 2024 Jul 19;4(7):e0003242. doi: 10.1371/journal.pgph.0003242 (PMC11259302; doi:10.1371/journal.pgph.0003242)
Supplement: S1 Table — (DOCX) [file pgph.0003242.s002.docx]

**Supporting information**

S1. Population treated and number of villages reached in Kapoeta East and Kapoeta North counties

during 2020 MDA

| **COUNTY** | **PAYAM** | **Total population treated** | **Total # of villages treated** |
| --- | --- | --- | --- |
| **Kapoeta East** | Jie Lopeat | 7184 | 9 |
|  | Jie Kasengor | 4279 | 6 |
|  | Katodori | 15423 | 96 |
|  | Kauto Central | 14892 | 89 |
|  | Kauto West | 17964 | 101 |
|  | Kauto East | 25455 | 369 |
|  | Lotimor | 12997 | 215 |
|  | Mogos | 30440 | 210 |
|  | Narus | 27458 | 164 |
|  | **Total** | **156092** | **1259** |
| **Kapoeta North** | Chumakori | 7022 | 71 |
|  | Karkamuge | 20352 | 130 |
|  | Lokwamor | 7677 | 48 |
|  | Lomeyen | 8164 | 75 |
|  | Najie | 7065 | 48 |
|  | Paringa | 7946 | 69 |
|  | **Total** | **58226** | **441** |
