# Supplementary material for: The cost of mass drug administration for trachoma in two counties of the Republic of South Sudan
Source: PLOS Glob Public Health. 2024 Jul 19;4(7):e0003242. doi: 10.1371/journal.pgph.0003242 (PMC11259302; doi:10.1371/journal.pgph.0003242)
Supplement: S2 Table — (DOCX) [file pgph.0003242.s003.docx]

**Supporting information**

S2. List of sub-areas and days of implementation in Kapoeta East county, South Sudan

| Sub-area | Days of implementation | Number of drug distributors |
| --- | --- | --- |
| Kauto West | 12 | 30 |
| Lotimor and Kauto East | 14 | 36 |
| Narus and Kotidori | 17 | 30 |
| Mogos | 13 | 30 |
| Jie | 5 | 27 |
