# Supplementary material for: The cost of mass drug administration for trachoma in two counties of the Republic of South Sudan
Source: PLOS Glob Public Health. 2024 Jul 19;4(7):e0003242. doi: 10.1371/journal.pgph.0003242 (PMC11259302; doi:10.1371/journal.pgph.0003242)
Supplement: S3 Table — (DOCX) [file pgph.0003242.s004.docx]

**Supporting information**

S3. The proportion of capital costs attributable to each county

|  | **Target population** | **Weights** |
| --- | --- | --- |
| North Kapoeta | 63,181 | 0.172392529 |
| East Kapoeta | 161,147 | 0.439697677 |
| South Kapoeta | 34,326 | 0.093660214 |
| Budi | 64,485 | 0.175950559 |
| Lafon | 43,356 | 0.118299022 |
| **Sum** | 366,495 | 1 |

S1 Table shows the weight that would be attributed to each county. The weight of Kapoeta North and Kapoeta East is calculated by dividing the target population of each county by the entire target population where The Carter Center Trachoma Control Program is operating.
