# Supplementary material for: The cost of mass drug administration for trachoma in two counties of the Republic of South Sudan
Source: PLOS Glob Public Health. 2024 Jul 19;4(7):e0003242. doi: 10.1371/journal.pgph.0003242 (PMC11259302; doi:10.1371/journal.pgph.0003242)
Supplement: S4 Table — (DOCX) [file pgph.0003242.s005.docx]

**Supporting information**

S4. List of costs and quantities included in costing analysis of MDA in Kapoeta East and Kapoeta North counties, South Sudan

| Cost item | Total cost | Quantity |
| --- | --- | --- |
| Landcruiser | USD $39,930 | 3 |
| Pick up | USD $32,500 | 1 |
| 4 Wheel Ural | USD $121,000 | 1 |
| 6 Wheel Ural | USD $125,978 | 1 |
| Motorbike | USD $4,287 | 3 |
| Vehicle maintenance (gross) | USD 23,531 | 1 |
| Motorbike maintenance (per bike) | USD 1,310 | 3 |
| Annual subscription/vehicle | USD $320 | 6 |
| Annual Airtime per vehicle | USD $600 | 6 |
| Vehicle satellite tracker | USD $170 | 6 |
| Honda generator | USD $661.85 | 14 |
| Tents | USD $200 | 15 |
| Laptops | USD $1,077 | 3 |
| Satellite phones | USD $345 | 7 |
| Hub security (Annual cost) | USD $38,217 | 1 |
| Hub electricity (Annual cost) | USD $16,800 | 1 |
| Internet (Annual cost) | USD $24,000 | 1 |
| Phone credit | USD $208 | 4 |
| Water (Annual cost) | USD $3,420 | 1 |
| Fuel per litre | USD $0.80 | 12,793 |
| Per diem for program staff (including technical advisor, senior program officers, drivers, program officers, data clerk) | USD $12 | 11 |
| Chiefs | USD $3.00 | 120 |
| Cooks per diem | USD $7.68 | 2 |
| Water porter day rate | USD $7.68 | 19 |
| Wood fetcher day rate | USD $5 | 1 |
| Social mobilisers | USD $802.24 | Total cost Kapoeta North |
| Social mobilisers | USD $2,816.67 | Total cost Kapoeta East |
| Per diem: drug distributors | USD $7.68 | 183 |
| Meals and refreshments (per person) | USD $5.00 | 183 |
| Shipping costs to Kapoeta | USD $2,500 | 1 |
| Shipping insurance | USD $4,761 | 1 |
| Police security per day | USD $65 | 1 |
| Printing | USD $300 | 1 |
| Torches | USD $14 | 30 |
| Batteries | USD $5.75 | 1,214 |
| Zithromax (Economic cost only) | USD $0.14 | Kapoeta East: 350,473  Kapoeta North: 118,496 |
| Powder Oral Suspension (Economic cost only) | USD $0.0367/ml | Kapoeta East: 516,734  Kapoeta North: 224,268 |
| Tetracycline eye ointment | USD $0.20 | Kapoeta East: 11,379  Kapoeta North: 3,916 |
